# A Case of Secondary Abdominal Pregnancy after in Vitro Fertilization Pre-Embryo Transfer (IVF-ET)

**DOI:** 10.3889/oamjms.2015.070

**Published:** 2015-06-25

**Authors:** Mariya Angelova Angelova, Emil Georgiev Kovachev, Ivan Kozovski, Yavor Dimitrov Kornovski, Stefan Vasilev Kisyov, Vilislava Robert Ivanova

**Affiliations:** 1 *Department of Obstetrics and Gynaecology, Medical Faculty, Trakia University of Stara Zagora, Stara Zagora, Bulgaria*; 2 *Department of Obstetrics and Gynaecology, Medical University of Varna, Varna, Bulgaria*; 3 *Varna OOD Medical Centre for Assisted Reproduction, Varna, Bulgaria*

**Keywords:** ectopic pregnancy, abdominal pregnancy, assisted reproductive technologies

## Abstract

The authors describe a rare case of secondary abdominal pregnancy after in vitro fertilization pre-embryo transfer (IVF-ET). Ultrasonography was applied to image ectopic gestational sac containing a yolk vesicle and located adjacent to the anterior uterine wall and left adnexa. Laparoscopy was done on the same day followed by sinistral salpingectomy due to tubal abortion indications. Intraabdominal examination showed chorionic structures penetrating pl. vesicouterina. Histological tests confirmed the EP diagnosis in the second material, i.e. indications of secondary abdominal pregnancy.

## Introduction

There are some specific risk factors for ectopic pregnancy (EP) in assisted reproductive technology (ART) such as reduced tubal contractility as a result of high levels of progesterone produced by multiple corpora lutea, hypervascular ovaries after hyperstimulation and follicular puncture, deep fundic embryo transfer (ET), large number of transferred embryos, uterine anomalies [1, 2].

It is believed that even at adequately performed ET embryos can migrate into the fallopian tubes due to the retrograde effect of uterine contractions. Upon performance of ET, the pressure exerted by the medium containing the embryos, may also contribute to the embryo migration in the fallopian tubes and at more than 80 microliters of medium, the risk is increased [3].

Genital chlamydiosis is one of the most common sexually transmissible diseases and a major cause of pelvic inflammatory disease (PID). Due to the extreme tropism of chlamydia to endosalpingauma, it can cause obturation, hydrosalpinx and diverticulitis in the fallopian tubes [4, 5]. Peritubal adhesions due to salpingitis, post abortion and puerperal infections, appendicitis are risk factors for ectopic pregnancy [6, 7].

## Description of the case

This is a 33-year-old patient with secondary sterility, male factor - oligoasthenozoospermia, moderate to severe form. Hysterosalpingography showed obturated left tube without hydrosalpinx, i.e. also presence of a tubal factor. Recommendations were given to apply diagnostic laparoscopy before IVF for full clarification of the status of the tube and the presence of adhesive syndrome, but the patient refused it.

**Figure 1:**
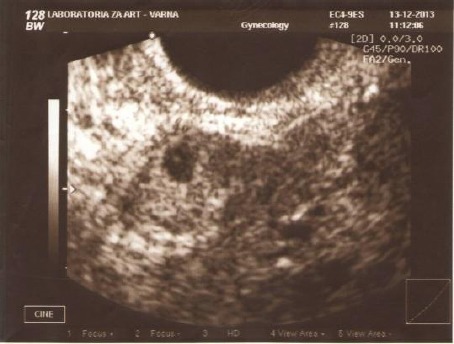
*Ultrasound visualization of ectopic gestational sac*.

Ovarian stimulation was carried out applying a short protocol with GnRH-antagonist and recombinant FSH preparation. Embryo transfer of two embryos was performed at 72 h after the puncture with 20 μl medium (BlastAssist®, Origio; Denmark) by Wallace catheter (Smiths Medical International, United Kingdom) and transabdominal ultrasound control, the embryos being transferred at 1-2 cm from the fundus. Serum level of β- HCG was measured on day 13 after ET (720 mIU/mL) and pregnancy quality test showed positive result (06.12.2013). The first transvaginal ultrasound scan (13.12.2013) was done in day 23 after the ET and no intrauterine pregnancy was identified in the presence of scarce genital bleeding (decidual reaction). An ectopic gestational sac containing a yolk vesicle and located adjacent to the uterine and left adnexa was imaged (Fig. 1).

**Figure 2:**
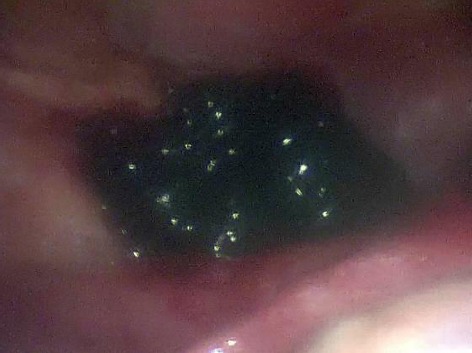
*Secondary abdominal pregnancy*.

**Figure 3:**
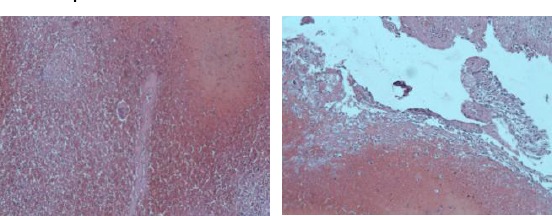
*Microscopic characteristics of the material from the abdominal pregnancy - presence of syncytiotrofoblast and decidual cells (our case)*.

Laparoscopy was done on the same day followed by sinistral salpingectomy due to tubal abortion indications. Intraabdominal examination showed coagula and chorionic structures penetrating pl. vesicouterina (Fig. 2). Histological tests confirmed the EP diagnosis in the second material (Fig. 3). No complications in the post surgery period. Values of the serum β-HCG progressively decreased.

## Discussion

The case of secondary abdominal pregnancy described above is rare in clinical practice.

The incidence of some rare forms and locations of ectopic pregnancy and of heterotopic pregnancy is on the increase after assisted reproductive technologies [8-10].

The authors describe cases of heterotopic interstitial pregnancy and bilateral tubal pregnancy after ART [11, 12].

In a hydrosalpinx diagnosis the recommendation is to sever the tubes by bipolar coagulation and incision, i.e. hydrosalpinx is not to communicate with the uterine cavity or unilateral or bilateral salpingectomy before the IVF procedure. In addition to high reduction of the risk of occurrence of EP, the performance of these procedures increases also the rate of clinical pregnancy after IVF-ET. Some patients with heterotopic, interstitial or corneal pregnancy after IVF-ET, have previously undergone bilateral salpingectomy and the absence of fallopian tubes does not complete eliminate the risk of these rare forms of ectopic pregnancy [13-15].

When considering ART procedures it is desirable that patients shall be informed about the risk of extra-uterine, heterotopic pregnancy, and some rare forms of ectopic pregnancy.
